# A Hypothetico-Deductive Approach to Assessing the Social Function of Chemical Signalling in a Non-Territorial Solitary Carnivore

**DOI:** 10.1371/journal.pone.0035404

**Published:** 2012-04-18

**Authors:** Melanie Clapham, Owen T. Nevin, Andrew D. Ramsey, Frank Rosell

**Affiliations:** 1 Centre for Wildlife Conservation, National School of Forestry, University of Cumbria, Penrith, United Kingdom; 2 Faculty of Art and Sciences, Department of Environmental and Health Studies, Telemark University College, Bø i Telemark, Norway; INRA-UPMC, France

## Abstract

The function of chemical signalling in non-territorial solitary carnivores is still relatively unclear. Studies on territorial solitary and social carnivores have highlighted odour capability and utility, however the social function of chemical signalling in wild carnivore populations operating dominance hierarchy social systems has received little attention. We monitored scent marking and investigatory behaviour of wild brown bears *Ursus arctos*, to test multiple hypotheses relating to the social function of chemical signalling. Camera traps were stationed facing bear ‘marking trees’ to document behaviour by different age sex classes in different seasons. We found evidence to support the hypothesis that adult males utilise chemical signalling to communicate dominance to other males throughout the non-denning period. Adult females did not appear to utilise marking trees to advertise oestrous state during the breeding season. The function of marking by subadult bears is somewhat unclear, but may be related to the behaviour of adult males. Subadults investigated trees more often than they scent marked during the breeding season, which could be a result of an increased risk from adult males. Females with young showed an increase in marking and investigation of trees outside of the breeding season. We propose the hypothesis that females engage their dependent young with marking trees from a young age, at a relatively ‘safe’ time of year. Memory, experience, and learning at a young age, may all contribute towards odour capabilities in adult bears.

## Introduction

Chemical signalling strategies in mammals are dependent on life-history patterns, social requirements and physical aspects of an individual's environment [1]. Indirect communication via chemical signals allow for multidimensional self-advertisement by a signaller, and the assessment of conspecifics by receivers [2]–[6]. Scent marking has been studied most prevalently in rodents, where it has primarily been shown to establish dominance between individuals [7]–[9] and signal attractiveness to potential mates [10]–[12]. In social and territorial species, scent marks often act as a means of territory defence [13] and for intra-sexual competition within and between groups [14]. Less is known about the functions of scent marking in solitary and non-territorial large mammals. Solitary species must maintain an effective communication system to uphold social organisation and facilitate reproductive opportunity [15]. Indirect communication via chemical signalling is a necessity for wide-ranging solitary carnivores [16] to achieve breeding rights, exercise dominance and maintain resource holding power over other individuals [3], [17].

Carnivores place scent marks onto, or in the vicinity of, conspicuous objects such as trees, or on the substrate in a conspicuous manner [18]–[20]. In solitary territorial carnivores, territory defence and the advertisement of the favourable traits of a male and oestrous state of a female (i.e. for mate attraction) appear to be the main functions of scent marking, particularly in polyestrous species [1], [16], [21]. Seasonally polyestrous solitary carnivores [22], [23] also show seasonal variation in scent marking frequencies with an increase by both sexes with the onset of oestrous, whereas annually polyestrous species show no seasonal variation [1]. Sexual dimorphism in the marking frequency of territorial solitary carnivores has been shown to be male biased [23], [24], with males increasing the frequency of their marks during pro-oestrous of females [23], [25]. Territorial solitary females appear to mark less than males [25], however some increase their marking frequency with the onset of oestrous [26]. Details on marking frequencies by females with dependent young are limited, but seem to take place at an even lower rate than lone females outside of oestrous [25], [27].

Scent marking in non-territorial solitary carnivores which operate a hierarchical social system may also function to attract mates via self-advertisement. Furthermore chemical signals may communicate dominance through the signalling of competitive ability, as displayed by other mammals [11], [28], [29]. Scent marking frequency outside of the breeding season would then be dependent on the density of individuals within the population i.e. an increased need to display dominance [30]. Consequently, it would be advantageous for subordinates to assess competitors through scent marks and regulate their behaviour accordingly, to avoid costly encounters [31]. Conversely, they may avoid areas where the potential for such encounters is elevated [32]. However, there is very little empirical evidence of the social function(s) of scent marking in wild non-territorial solitary carnivores.

Brown bears *Ursus arctos* are a predominantly solitary species which display a dominance hierarchical social system, promiscuous mating system, and sex-biased dispersal where males disperse further from the natal range than females [33]–[36]. Females exhibit kin-related spatial structure which may form multigenerational matrilineal assemblages [34], [37]. They are not strictly territorial but have home ranges which overlap both inter- and intrasexually [34], [38]. Due to the solitary and wide-ranging nature of their ecology, bears are thought to rely heavily on chemical signals as a means of communication (Rosell et al. unpublished). For example, Rosell et al. [39] recently found that the anal gland secretion (AGS) of wild brown bears ≥3 years of age contains a chemical code for sex. Jojola [40] also found that captive subadults (1–3 yrs of age) were able to distinguish the sex of adult bears from their AGS, and investigated that of males more intensively. However, with the exception of giant pandas *Ailuropoda melanoleuca*, whose chemical communication abilities have been studied extensively in captivity [41], [42], no study has yet been able to demonstrate the function(s) of chemical signalling in wild bear populations.

As with other solitary carnivores, bears seem to deposit their scent marks on the substrate and/or conspicuous objects (trees) [43]–[46]. Substrate marks by female giant pandas have been found to contain AGS and vaginal secretions, and contain information relating to age sex class and individual identification [46], [47]. Male giant pandas are said to leave such marks on trees and rocks, rather than the substrate [47]. Brown bears claw, bite, urinate and also rub various parts of the body against trees [43], [45], [48], [49], yet little is known concerning sexual differences in this species. ‘Traditionally used trees’ can be repeatedly marked over many generations [44] and are said to emphasize the link between scent marking and intraspecific communication in bears [45] rather than a response to external stimuli as previously suggested [43]. However the detailed use of such marking trees by wild bears has still to be examined, and may provide an insight into their function(s).

Burst and Pelton [43] indicated an aspect of seasonality in the marking behaviour of black bears *U. americanus*; detecting more fresh marks on trees prior to and during the breeding season than other times of year. Through genetic analysis of hairs from marking trees for population monitoring, it has been indicated that adult male brown bears may increase their marking frequency during the breeding season [50], [51]. Conversely it is important to know which individuals do not partake in scent marking behaviour, and which purely investigate marks; knowledge unattainable through genetic analyses or visual assessments of marking trees.

Dependent young are at risk of infanticide, particularly during the breeding season [52], [53] when young are vulnerable to sexually selected infanticide (SSI) [54]. Male subadult bears, which have departed from their mother but not yet reached sexual maturity, are also at risk of aggression from adult males [44]. In line with the communicating dominance and self-advertisement hypotheses, subadults and females with dependent young would be unlikely to display high levels of scent marking, particularly during the breeding season. They may however investigate scent marks to assess potential competitors and use the information gained to avoid potentially fatal encounters [42]. As female brown bears with dependent young can display seasonal spatial segregation, seemingly to avoid SSI [55], they may avoid marking trees altogether. To date, no studies have been able to provide a thorough insight into the social function(s) of seasonal age and sex differences in tree marking and investigation by bears in the wild.

In this study, we investigate the function(s) of chemical signalling in a solitary, non-territorial carnivore, the brown bear. We follow a hypothetico-deductive approach to test the following hypotheses (Table 1), that bears use scent marking for; (1) self-advertisement for mate attraction, (2) communicating dominance, and (3) competitor assessment. We also test for evidence of the infanticide avoidance hypothesis displayed by females with dependent young (4). These hypotheses are not mutually exclusive and there is a possibility that scent marking in brown bears will prove to be multi-functional. For a hypothesis to be supported, at least two-thirds of its related predictions should be satisfied. The hypotheses and predictions tested are summarized in Table 1.

**Table 1:** Hypotheses and predictions tested with summary of outcomes.

Hypothesis	Outcome which supports hypothesis	Prediction supported?	Hypothesis supported?
**1. Self-advertisement for mate attraction**			
1.1 AM self-advertise	1.1.1 AM will scent mark at a higher frequency than expected* during the BS	Y	P
	1.1.2 AF will investigate at a higher frequency than expected* during the BS	N	
	1.1.3 During the BS AM will scent mark and AF will investigate more than any other age sex class	Y (AM) N (AF)	
	1.1.4 During the NON-BS scent marking and investigation by AM and AF respectively will be less than expected*	N (AM) Y (AF)	
1.2 AF self-advertise	1.2.1 AF will scent mark at a higher frequency than expected* during the BS	N	N
	1.2.2 AM will investigate at a higher frequency than expected* during the BS	N	
	1.2.3 During the BS AF will scent mark and AM will investigate more than any other age sex class	N	
	1.2.4 During the NON-BS scent marking and investigation by AF and AM respectively will be less than expected*	N (AM) Y (AF)	
1.3 SUB avoid self-advertisement	1.3.1 SUB will scent mark at a lower frequency than expected* during the BS	Y	Y
	1.3.2 During the BS SUB will scent mark less than AM and AF	Y (AM) N (AF)	
	1.3.3 During the NON-BS SUB will scent mark at an expected frequency*	Y	
1.4 SUB utilise self-advertisement of others	1.4.1 SUB will investigate at a higher frequency than expected* during the BS	N	N
	1.4.2 SUB will investigate at a frequency equivalent to AM and AF during the BS	Y	
	1.4.3 During the NON-BS SUB will investigate less than expected*	N	
**2. Communicating dominance**			
2.1 AM communicate dominance	2.1.1 AM will scent mark at a higher frequency than expected* during the BS	Y	Y
	2.1.2 AM will scent mark at a higher frequency than expected* during the NON-BS	Y	
	2.1.3 AM will mark at a higher frequency than any other age sex class in both the BS and NON-BS	Y	
2.2 AF do not communicate dominance	2.2.1 AF will scent mark at a lower frequency than expected* during the BS	Y	Y
	2.2.2 AF will scent mark at a lower frequency than expected* during the NON-BS	Y	
	2.2.3 AF will scent mark at a frequency lower than AM in both the BS and NON-BS	Y	
2.3 SUB avoid communicating dominance	2.3.1 SUB will scent mark at a lower frequency than expected* during the BS	Y	P
	2.3.2 SUB will scent mark at a lower frequency than expected* during the NON-BS	N	
	2.3.3 SUB will be scent mark at a frequency lower than adults in both the BS and NON-BS	Y (AM) N (AF)	
**3. Competitor assessment**			
3.1 AM assess competitors	3.1.1 AM will investigate at a higher frequency than expected* during the BS	N	N
	3.1.2 AM will investigate at a higher frequency than expected* during the NON-BS	N	
	3.1.3 AM will investigate at a higher frequency than any other age sex class in both the BS and NON-BS	N	
3.2 AF assess competitors	3.2.1 AF will investigate at a higher frequency than expected* during the BS	N	N
	3.2.2 AF will investigate at a higher frequency than expected* during the NON-BS	N	
	3.2.3 AF will investigate at a higher frequency than any other age sex class in both the BS and NON-BS	N	
3.3 SUB assess competitors	3.3.1 SUB will investigate at a higher frequency than expected* during the BS	N	N
	3.3.2 SUB will investigate at a higher frequency than expected* during NON-BS	N	
	3.3.3 SUB will investigate at a frequency equivalent to adults in both the BS and NON-BS	Y (BS) N (NON-BS)	
**4. Infanticide avoidance**			
4.1 F+Y avoid chemical communication	4.1.1 F+Y will scent mark at a lower frequency than expected* during the BS	N (>1 yr)	N
	4.1.2 F+Y will scent mark at a lower frequency than expected* during the NON-BS	N (all ages)	
	4.1.3 F+Y will investigate at a lower frequency than expected* during the BS	N (>1 yr)	
	4.1.4 F+Y will investigate at a lower frequency than expected* during the NON-BS	N (all ages)	
	4.1.5 F+Y will scent mark and investigate at a lower frequency than any other age sex class during both the BS and NON-BS	N (all ages)	
4.2 F+Y avoid marking trees	4.2.1 F+Y will be present on bear trails containing active marking trees at a lower frequency than their proportion in the observed population would suggest during the BS and NON-BS	N (all ages - BS+NON-BS)	N

* in relation to their presence on trails containing active marking trees.

AM – adult male, AF – adult female, SUB – subadult, F+Y – female with dependent young, BS – breeding season, NON-BS – non-breeding season, Y/N – yes or no, P – partially supported.

## Materials and Methods

### Ethics statement

Ethical clearance was approved by the University of Cumbria Ethics Committee, ref 09/10. Due to the non-invasive, observational nature of the work, research permits were not required. Research was carried out on crown land; therefore permission was sought from the Ministry of Environment, Government of British Columbia.

### Study site

Glendale Cove is an estuarine inter-tidal zone of Knight Inlet, British Columbia, Canada (N 50°41′W 125°44′). Situated in the Pacific mid-coast of the Province, it has a mild, hyper-maritime climate. During spring/summer brown bears are attracted to the tidal marshes to feed on herbaceous vegetation (*Carex* spp.). This coincides with the breeding season when adult males, lone adult females and courting pairs are often seen in this area. At this time of year, females with dependent young are often viewed towards the far north of the estuary feeding along the inter-tidal zone. The breeding season for brown bears is typical of other areas of its range, beginning in late May and continuing until mid/late July [56], [57]. Approximately 40 brown bears utilise the Glendale spawning channel as a primary energy resource during hyperphagia, due to the return of five anadromous salmoniod species (*Oncorhynchus* spp.) [56], [58]. Breeding season data was collected between 1^st^ June and 31^st^ July in 2009 and 2010. Non-breeding season data was collected between 1^st^ August and 5^th^ October 2010.

### Location of marking trees and monitoring procedure

Twenty-one traditionally used trees were monitored which showed signs of recent marking activity. Marking trees were identified by sampling the forest along wildlife trails and a disused logging road which ran parallel to the west shoreline of the estuary. Marking signs included claw and bite marks, remnants of hair displaying evidence of rubbing, and wounds on trees due to bark stripping. Scars caused by clawing and biting indicated that the tree was traditionally used. Fresh marks were indicated by fresh oozing sap from wounds, lacerated bark which left fragments exposed, the colour of exposed wood, and any remnants of hair loosely attached. Most marking trees were located at forest edges bordering the estuary. Cameras allowed for the recording of marking behaviour not easily detected by visual observation of trees, such as cheek rubbing. All cameras were stationed in different locations to maximise the capture rate of different individuals. Seven ‘Reconyx’ (Reconyx Inc., Wisconsin, USA) model RC55 digital passive still-image trap cameras were deployed in 2009, and supplemented by a further 10 ‘Reconyx’ model PC85 cameras in 2010. Cameras were set up to record images both day and night via the infrared flash. Cameras were positioned opposite marking trees, facing along a trail so to capture the behaviour of the target on approach and departure. Cameras were initially checked after 2–3 days to ensure correct positioning and function and then left for two weeks before any data was assessed. Cameras that recorded little or no activity were relocated. Subsequently, easily accessible cameras (*n* = 9) were checked approximately every week, with less accessible ones (*n* = 8) checked every two weeks.

Scent marking and investigatory behaviour was documented outside of the breeding season to assess for potential seasonal differences. Camera traps were repositioned facing marking trees in areas of high activity during the pink salmon (*O. gorbuscha*) run from August onwards [59]. Camera protocols remained as previously described.

### Direct visual observations

Direct observations were used to supplement data and knowledge of the sample population. This included identification of individuals, recognition of those individuals on images, assignment of individuals to the correct age sex class, and monitoring and documentation of mating behaviour. Direct visual observations during the breeding season were conducted primarily by boat, but also occasionally on land. Photographs, written descriptions, and identification sheets were used to document distinguishing characteristics of individuals. Observations were conducted during daylight hours between 0730 and 1800 hrs. Observations were usually in three hour blocks, based around high tide. During the non-breeding season, direct visual observations were conducted from viewing platforms located at an artificial weir, where bears congregate due to increased fishing opportunities. Observations were generally conducted during daylight hours, in five hour blocks.

### Classifying and categorising images

A total of 1,265 trap nights during the breeding season and 1,024 trap nights during the non-breeding season were carried out. A trap night was defined as a 24-hour monitoring period by each camera, as in Ríos-Uzeda et al. [60]. Camera trap images from the breeding and non-breeding seasons were analysed separately. Each occasion where a brown bear was captured on an image, or set of images, was classified as an ‘event’ [61] and given a unique ID. The age sex class of the individual(s) captured was assessed (Table 2) and their behaviour recorded (Table 3) for each event. Courting pairs were classified as individuals, but their association was noted. Where images of an individual were separated by less than five minutes on a single camera they were not considered independent events.

**Table 2:** Classification to age sex class of individuals captured on images and directly observed.

Age class	Sex class	Identifiers	Sexes classified separately?	Behaviour classified separately?	Termed hereafter
Adults	Male	Observation of genitals	Yes	Yes	Adult males
		Size/weight			
		Urination pattern			
		Observed breeding/courting			
	Female	Observation of genitals	Yes	Yes	Adult females
		Size/weight			
		Urination pattern			
		Drooping mammary glands			
		Observed breeding/courting			
	Female with dependent young	Presence of young	No	No	Females with dependent young (all ages)Females with cub(s) (<1 year of age)Females with yearling(s) (1–2 years)
		Swollen mammary glands			
		Lactating			
Subadults	Male/Female	Independent	No*	No*	Subadults
		Size/weight			
		Subordinate behaviour			
		Not observed breeding/courting			

* noted if known.

**Table 3:** Behavioural categories developed prior to classification of images.

Behavioural category	Classification	Description
Communication	Scent marking	Direct contact with tree through
		rubbing any body part
		clawing
		biting/licking
		Rolling on ground in direct vicinity of tree
		Sitting next to/against tree
Investigation	Investigating a scent mark	Direct contact with tree using
		nose - sniffing
		Head angled towards tree with
		neck stretched
		nose lifted/twisted
		Changing course of direction to approach tree
		Hesitating and visibly angling body/head towards tree
		Smelling ground in direct vicinity of tree
Locomotion	Using trail	No direct contact with tree
		Walking/trotting/running past showing no visible interest in tree
		No hesitation or change of direction when in direct vicinity of tree

Events were classified under the locomotion category unless a communication or investigation descriptor was satisfied. Locomotion included all behaviours unrelated to a marking tree.

All captured bears, whether observed through direct visual observations or through camera traps, were assigned a unique identifier. Individuals were identified by comparing camera trap and other images, capture timings in relation to spatial distribution of cameras, and information on identification sheets. Discounting dependant cubs, 25 different individuals were identified during the breeding season, and 51 during the non–breeding season. Eleven out of the 25 individuals identified during the breeding season were also captured during the non-breeding season. As in Negrões et al. [62], time-stable and time-variable parameters were used for individual identification of bears. However instead of using coat colouration as a time-stable parameter, it was used as a time-variable parameter, as coat colouration in bears can vary temporally, particularly in younger bears (authors' pers. obs.). Coat colouration can also appear darker when wet (authors' pers. obs.). Scar patterns were used as a time-stable parameter for individuals within a season, supported by additional parameters between seasons. Additional parameters typically included a combination of: size, presence/absence of cubs, behaviour around boats/people, behaviour around other bears, individual stereotyped behaviours, and distinguishing morphological features such as a short snout or pale claws. To increase the reliability of the identification, new individuals were only assigned a unique identifier when captured on camera at least twice or captured once and directly viewed on a separate occasion. Identification of individuals was enhanced when subjects were captured on cameras from different profiles and in colour photographs captured during daylight hours. The individuals captured on camera traps were combined with individuals only viewed by direct observations to give the number of individuals of each age sex class observed in the area at that time.

### Data analysis

Data from breeding and non-breeding seasons were analysed separately using χ^2^ goodness-of-fit tests, unless otherwise stated. Firstly, a comparison between the number of individuals of each age sex class in the population, and the number present on trails containing active marking trees was made. For the breeding season, the proportions of each were calculated using the maximum number of individuals in each age sex class observed in either of the two years. Comparisons of the general population with age sex ratios recorded in 2005/2006 (Nevin, unpublished data) yielded no significant difference (χ^2^ = 0.156, df = 3, *p* = 0.984), indicating a stable age structure. By using the maximum number over two years rather than the mean, individuals which may have been present but not captured on cameras or directly observed, could be included in analysis. Secondly, a comparison was made between the frequencies of events per individual, within age sex classes. As data were found to be non-parametric in all cases, Kruskal-Wallis tests were used to identify differences in the distributions of each age sex class: present on trails, marking, and investigating trees. Thirdly, frequencies of scent marking and investigation of scents by each age sex class were examined in relation to their frequency on trails. This allowed for the assessment of behaviours associated with marking trees at a larger population level, rather than individual. Since there was no significant difference between the breeding seasons of 2009 and 2010 (χ^2^ = 0.181, df = 3, *p* = 0.981), behavioural frequency data for each age sex class were pooled in further analyses. Events which captured the same individual on different cameras were considered independent for this part of analysis, due to the spatial distances of the cameras (minimum distance between two cameras = approx. 100 m) and their focus on different marking trees. Outcomes were analysed to support or reject the hypotheses and predictions outlines in Table 1; it is important to note that multiple predictions can be tested through post-hoc subdivision of individual tests and that this does not represent a repeated reanalysis of the dataset with the associated risk of Type I error [59], [63]. Association χ^2^ tests were also applied to both scent marking and investigatory behaviour by each age sex class in both the breeding and non-breeding seasons. This allowed for the proportion of scent marking/investigating events to be analysed in relation to the total capture events of each age sex class.

## Results

A total of 1,050 events were captured during the breeding and non-breeding seasons. Classification of images revealed 733 independent camera trap events assigned to age sex class; 694 were also identified to the individual level, and assigned to their unique identifier. A total of 159 behavioural events during the breeding season and 574 during the non-breeding season were documented, and assigned to an age sex class. There were 39 events where individuals could not be identified, all from the non-breeding season: of these, 8 were adult males, 2 adult females, 22 females with dependent young, and 7 subadults. The individual(s) in 12 events during the breeding season and 67 during the non-breeding season could not be identified to an age or sex class. The individual(s) captured in 25 events during the breeding season and 252 events during the non-breeding season could not be sexed but were identified as adults.

Table 4 displays the number of individuals included in analyses for each age sex class and season. No significant difference was found between the proportion of individuals within each age sex class captured on trails and the general population observed at that time (breeding season: χ^2^ = 0.378, df = 3, *p* = 0.945; non-breeding season: χ^2^ = 0.761, df = 3, *p* = 0.859). Therefore the presence of bears on trails was said to be representative of the observed population in the area.

**Table 4:** Number of individuals used in comparisons between presence on trails and the general population.

	Breeding season					Non-breeding season				
	am	af	f+y	sub	*n*	am	af	f+y	sub	*n*
Individuals present in the general population (max. across years)	7	5	3	6	21	21	12	10	9	52
Individuals present on trails (max. across years)	7	5	2	5	19	17	9	10	9	45

am = adult males, af = adult females, f+y = females with young, sub = subadults, *n* = total individuals.

The median number of capture events per individual varied by age sex class and according to season. Events during the breeding season consisted of a median capture of 5 events per adult male (*n* = 7: (min-max) 1–32), 4 per adult female (*n* = 7: 1–11), 11 per female with young (*n* = 2: 3–19) and 2.5 per subadult (*n* = 8: 1–8). No significant difference was found between age sex classes, in the frequencies of individuals: present on trails (*H* = 4.173, df = 3, *p* = 0.243), marking (*H* = 4.987, df = 3, *p* = 0.173) or investigating (*H* = 4.246, df = 3, *p* = 0.236). The non-breeding season consisted of a median of 6 events per adult male (*n* = 17: 1–46), 4 per adult female (*n* = 9: 2–89), 15.5 per female with young (*n* = 10: 3–41) and 3 per subadult (*n* = 9: 1–21). The frequencies of marking by individuals between age sex classes was significantly different during the non-breeding season (*H* = 8.203, df = 3, *p* = 0.042). There were insufficient data to conduct pair-wise comparisons to identify the age sex classes contributing to this significance. No significant difference was found between age sex classes in the frequencies of individuals present on trails (*H* = 6.077, df = 3, *p* = 0.108) or investigating (*H* = 4.818, df = 3, *p* = 0.186).

**Figure 1:**
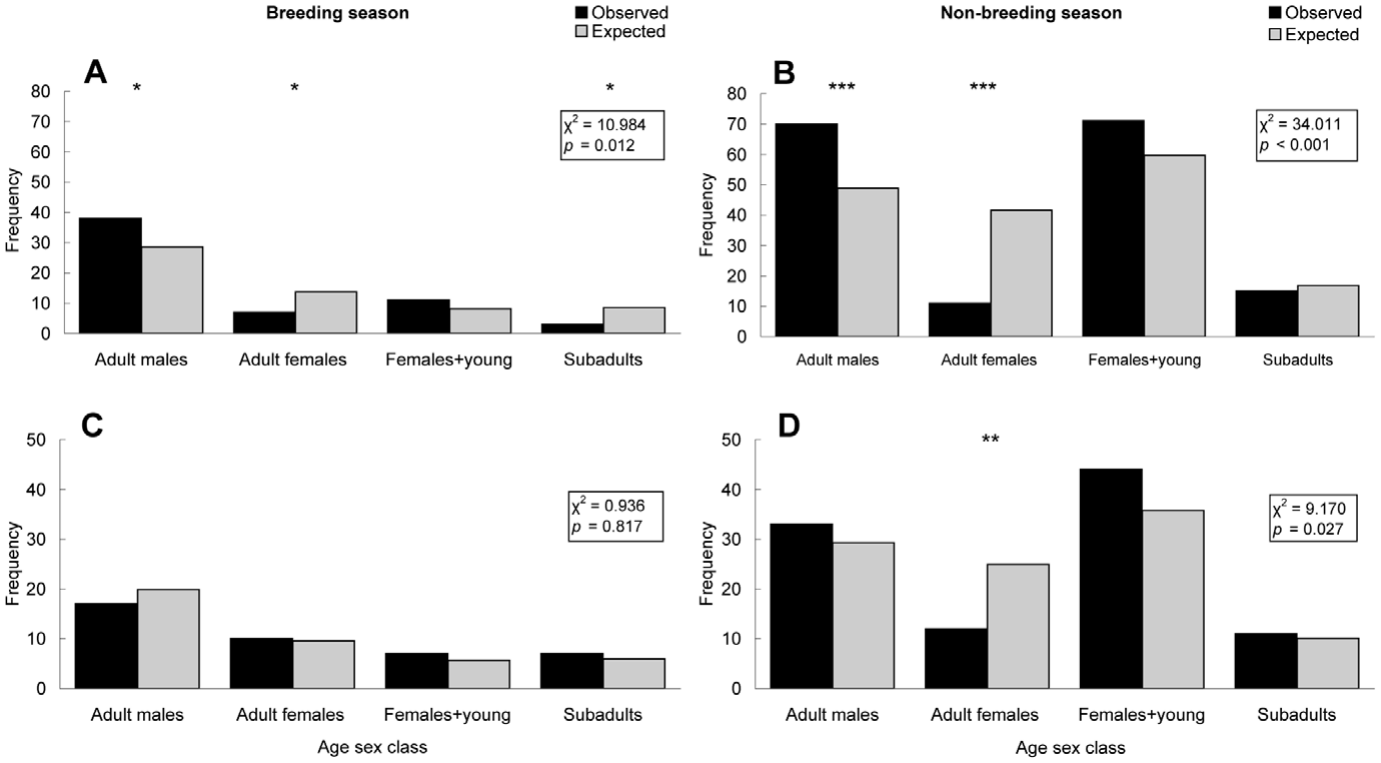
Behavioural frequencies in relation to trail use. Total observed events of marking (A, B) and investigation (C, D) by each age sex class, compared to their expected frequency in relation to their presence on trails containing active marking trees. Comparisons during the breeding (A & C) and non-breeding season (B & D). *** indicates *p*<0.001 in subdivided testing, ** *p*<0.01 and * *p*<0.05.

Comparison of frequencies of events at a population level revealed variation in marking and investigatory behaviour as a product of age sex class and season. When using trails containing marking trees, adult males marked trees more than expected across both seasons (Fig. 1 A & B). When using trails, adult females marked less than expected across both seasons (Fig. 1 A & B). No females with young (<1 year of age) were captured on cameras during the breeding season. Females with young (>1 yr during the breeding season; all ages during the non-breeding season) marked and investigated as expected when using trails across both seasons (Fig. 1 A–D). Subadults marked less than expected when using trails during the breeding season (Fig. 1 A), but as expected during the non-breeding season (Fig. 1 B). When using trails, all brown bears investigated marks as expected (Fig. 1 C & D), except adult females, who investigated marks less than expected outside of the breeding season (Fig. 1 D).

Results of association tests and the frequency of marking and investigating by each age sex class, within each season, are displayed in Table 5. Adult males were associated with scent marking behaviour significantly more than expected across both seasons. During the non-breeding season, they were more likely to mark a tree than not, when passing on a trail. Adult females were associated with scent marking behaviour significantly less than expected across both seasons. During the non-breeding season, they were more likely not to mark a tree than to mark it, when passing on a trail. Adult females were associated with investigating marks significantly less than expected during the non-breeding season. Subadults were associated with scent marking significantly less than expected during the breeding season. Related outcomes of hypotheses and predictions are outlined in Table 1.

**Table 5:** χ^2^ for association tests displaying total behavioural events per age sex class for the breeding and non-breeding season.

	Breeding season			
	Scent marking		Investigating	
	Marking	Non-marking	Investigating	Non- Investigating
Adult males	38+	39	17	60
Adult females	7−	30	10	27
Females with young	11	11	7	15
Subadults	3−	20	7	16
	χ^2^ = 17.464, df = 3, *p*<0.001		χ^2^ = 1.261, df = 3, *p* = 0.738	

	Non-breeding season			
	Scent marking		Investigating	
	Marking	Non-marking	Investigating	Non-investigating
Adult males	70+	98−	33	135
Adult females	11−	132+	12−	131
Females with young	71	134	44	161
Subadults	15	43	11	47
	χ^2^ = 47.967, df = 3, *p*<0.001		χ^2^ = 11.105, df = 3, *p* = 0.011	

+/− indicates significantly more/less than expected (*p*<0.05).

## Discussion

To our knowledge we are the first to publish data using camera traps to assess scent marking behaviour in wild Ursids, and the first to assess the proportional rate of chemical signalling by different age sex classes of wild brown bears across seasons. Other studies using camera trap images to visually identify carnivores with a relatively uniform pelage report a lower capture success and/or lack the ability to identify between individuals/genders [60], [64], [65]. Studies using camera traps to capture bears report effort between 493 and 1,200 trap nights [60], [66], [67], much lower than the sampling intensity employed in this study.

The presence of different age sex classes on trails was found to be representative of their proportion in the general population, indicating that this study does not just represent a subset of individuals. The frequency of behaviours shown by each age sex class can therefore be attributed to the behaviour of the population at that time.

### Self-advertisement for mate attraction

A significant male bias in scent marking behaviour was found during the breeding and non-breeding seasons. Adult males scent marked more than expected in relation to their presence on trails, and were associated with marking more than any other age sex class across both seasons. This continued activity is inconsistent with the self-advertisement for mate attraction hypothesis which predicted that adult males would mark less outside of the breeding season. We also found evidence inconclusive with the male-biased self-advertisement hypothesis in the behaviour of adult females. Adult females did not investigate scent marks more than expected in relation to their presence on trails during the breeding season, nor was investigation positively associated with the age sex class over others. Outside of the breeding season adult females did investigate marking trees less than expected, which could highlight a change in behaviour consistent with the self-advertisement hypothesis. However, without an increase in investigatory behaviour during the breeding season in adult females, tree marking cannot be related to self-advertisement by adult males. Instead, this result seems to represent a higher frequency of adult females on trails outside of the breeding season, rather than a change in the frequency of investigation. Bellemain et al. [68] suggest that under the influence of infanticide, females may mate with geographically close males, with paternity being decided through cryptic female choice. Correlates of male fitness via chemical signals on marking trees may therefore not be an integral part of female mate choice in brown bears.

Concerning evidence for female-biased self-advertisement, adult females marked less than expected in relation to their presence on trails, and were associated with marking behaviour less than expected during both the breeding and non-breeding seasons. Adult males investigated as expected, and were not associated with investigating significantly more than other age sex classes during both seasons. Thus adult female biased self-advertisement to advertise oestrous state is unlikely to be the basis for chemical signalling via marking trees in the species. However, this does not rule out the use of other means of chemical signalling by adult females to advertise oestrous, such as substrate marks seemingly used by female giant pandas [46].

Subadults avoided self-advertisement during the breeding season; they scent marked less than expected and were associated with marking less than expected. Their scent marking behaviour in relation to their frequency on trails and association with marking was as expected during the non-breeding season; suggesting an increase in marking by subadults outside of the breeding season. Subadults do not appear to investigate marking trees specifically to eavesdrop on the self-advertisement of adults. Subadults investigated marking trees as expected across both seasons. As no one age sex class were associated with investigating more than others during the breeding season, subadults investigated at an equivalent frequency to adults. Subadults are vulnerable individuals, particularly those who have recently separated from their mothers [69]. The low marking frequencies we observed in subadults are as expected, particularly during the breeding season when adult males are roaming to mate [55], and subadults are at an increased risk of intraspecific competition [69]. Swenson et al. [52] found predation on subadults to be at its highest during the breeding season. Subadults would therefore benefit by selecting a strategy which would allow them access to productive food resources [70], [71] but avoid the cost of intraspecific competition. By not necessarily avoiding productive areas where adults accumulate, but selecting not to mark trees in these areas, subadults may be able to avoid signalling information to adult individuals which may convey competition and prove costly.

### Communicating dominance

We found extensive support for communicating dominance hypotheses in the behaviour of adult males and adult females across seasons. Adult males scent marked more than expected in relation to their presence on trails, and were associated with marking more than any other age sex class, across both seasons. In addition, the lack of investigation bias by adult females during the breeding season suggests this form of signalling is aimed more within, than between, sexes. This behaviour concurs with Gosling's hypothesis [3] of males advertising their competitive ability to other males in order to protect the holding of a resource, in this case an oestrus female. According to Dahle and Swenson [55] outside of the breeding season adult males seem to concentrate their movement patterns around food resources. Social hierarchy is still maintained during this period in aggregations that form around key food resources [72], [73]. Tree marking during the non-breeding season may function to reduce physical conflict in aggregations around food, providing an energetic benefit to both signaller and receiver. Thus we find strong evidence to support the hypothesis that adult males utilise scent marking to communicate dominance across seasons.

We found no evidence to suggest that adult females may scent mark to communicate dominance to other females. Adult females scent marked less than expected from their presence on trails, and were associated with marking less than expected and less than adult males, across seasons.

We found partial support for the hypothesis that subadults avoid communicating dominance. During the breeding season their scent marking behaviour was significantly less than their frequency on trails would expect, but in proportion during the non-breeding season. Association tests showed an increased association between subadults and scent marking behaviour outside of the breeding season, compared to during. Their association with marking was equivalent to adult females during the breeding season, higher than adult females during the non-breeding season, and continuously less than adult males across seasons. The function of marking behaviour in subadults is relatively unclear. Its under-representation during the breeding season suggests a link to the marking-bias by adult males. As sexes were pooled in analysis, further assessment of scent marking behaviour in subadults may require the sexes to be split to highlight sexual differences consistent with that of adult bears.

### Competitor assessment

We found little evidence to support the competitor assessment hypothesis as a sole function of scent marking in brown bears. Neither adult males nor females investigated marking trees more than expected from their presence on trails during the breeding season. During the non-breeding season adult males investigated marks as expected, whereas adult females investigated less than expected. Adult females also investigated marking trees less than any other age sex class during the non-breeding season. We found little evidence, within predictions, to support the hypothesis of subadults overtly utilising the scent marks of adults to gain information on future competitors/conspecifics in the area. Subadults investigated scent marks in relation to their presence on trails across both seasons. Jojola [40] found captive subadult brown bears to investigate the AGS of adult males more intensively than females. Although we did not document a significant bias to investigating in either season, subadults investigated trees more often than they marked during the breeding season. Investigating marks may therefore provide a benefit to subadults during the breeding season. As stated under the self-advertisement hypothesis, subadults are at an increased risk of competition with adult males during the breeding season [52], [69]. It would benefit subadults to utilise the dominance advertisements of adult males left on marking trees to collect information on individuals within the area at that time. The similarity between the scent left as a scent mark and the marker itself may then be ‘matched’, influencing the subadult's response during potential future agonistic encounters [74].

It is unlikely that competitor assessment is the sole primary function behind chemical signalling in brown bears as no related hypotheses were satisfied in this study. It would be advantageous for adult males to assess competitors via scent marks and regulate their behaviour according to their own competitive ability [74], [75]. However as the study site is a high density area with productive food resources, a high concentration of dominant males may shape the pattern of communication. Larger males would then assess competitors and communicate their own dominance at marking trees, rather than purely assessing competitors. Harmsen et al. [30] state that marking frequency is affected by density, with higher densities promoting dominance related marking. Therefore competitor assessment may be a requisite for communicating dominance, but is masked by subsequent dominance related marking activities which flow from initial assessment behaviours.

### Infanticide avoidance

We found conflicting evidence for the infanticide avoidance hypothesis by females with dependent young. We documented a complete avoidance of trails containing active marking trees by females with young (<1 year of age) during the breeding season. Despite females with cubs being observed bordering areas containing marking trees, we did not capture any events containing cubs of this age or their mothers. We did however capture females with yearlings scent marking and investigating trees during the breeding season. Females with yearlings contradicted the infanticide avoidance hypothesis by scent marking and investigating in proportion to their presence on trails. During the non-breeding season we documented a shift in behaviour by females with dependent young; for the first time we captured females with cubs (<1 yr) on camera traps. We predicted that females with dependent young of all ages would avoid marking trees and scent marking behaviour, but failed to make an informed prediction as to whether this behaviour would change over time. The proportion of different age sex classes present on trails was found to be representative of the general population in the area at that time. However, a low sample size for females with young during the breeding season could have increased the chance of a Type II error here. Swenson et al. [53] found that young cubs were mostly killed during the breeding season. Vulnerable females which potentially provide reproductive opportunities to males avoid high-quality, male dominated areas [32], [70]. The age at which cubs, and therefore females with cubs, are most vulnerable is at the time of their initial emergence from the den, with the breeding season occurring shortly after. If females adopt an avoidance strategy, they risk decreased body condition possibly leading to increased mortality of cubs. However if they change their strategy outside of the breeding season, when infanticide is less prevalent, to take advantage of preferred habitats and continue to use this resource the following year, female body condition should have recovered after 1.5 years [76]. The number of females with young in the study area dramatically increased during the non-breeding season; resulting in an increase in marking frequency, which was as expected from their presence on trails. At this time of year, females with young appear to take advantage of areas of preferred habitat, but instead of residing in one area of the study site, move around more than any other age sex class (authors' pers. obs.). They held the highest frequency on trails of all age sex classes, the highest frequency of investigation of all age sex classes, and an observed marking frequency equivalent to that of adult males; despite their sample size being half that of males in the general population. Yet, unlike adult males, they were not associated with marking behaviour during either season. Females with young seem to have an involvement with marking trees which cannot be attributed to the marking hypotheses outlined here. Possible functions of this increase in presence on trails during the non-breeding season are to exploit a resource as much as possible, to navigate young around the natal range, or to keep mobility high to avoid intraspecific competition with adult individuals. We propose an alternative hypothesis, that memory, experience and learning of marking trees may be required early on in life, for bears to acquire advanced olfactory capabilities. Neurological research on captive small mammals has revealed the importance of memory, experience, and learning in odour discrimination [77]–[80]. Social dominance hierarchies seem to rely on learnt recognition of individuals or groups through olfactory signals and cues [81]–[83]. If cubs are able to recognise the scent of an adult male, they may be able to avoid SSI, or intraspecific competition as a subadult. The safest way for a female to provide her cubs with an olfactory stimulus as such, would be to provide them with the opportunity to investigate scent marks left on environmental objects, outside of the breeding season. This scent could then be matched to that of an adult male [74]. Based on field observations and camera trap images we have documented what could be interpreted as imitation behaviour by young cubs of their mothers marking/investigating trees (unpublished data). Further evidence is needed to develop this hypothesis and assess its application among, and outside of, the Ursidae.

In conclusion, we have assessed the social functions of chemical signalling via marking trees in a non-territorial solitary carnivore, the brown bear. Foremost, scent marking seems to facilitate the communication of dominance between adult males. However adult females and subadults may utilise such signals to their advantage. Chemical signalling via marking trees does not appear to function to advertise oestrous state in adult females. The function of marking by subadult bears is somewhat unclear, but may be a product of the behaviour of adult males. Subadults investigate trees more often than they scent mark during the breeding season, which could be a result of an increased risk from adult males. Females with young cubs display a shift in behaviour outside of the breeding season, which we hypothesize relates to the engagement of young with marking trees at a relatively ‘safe’ time of year. The seasonal and age sex differences in chemical signalling behaviour observed in brown bears can most probably be applied to other monoestrous or seasonally polyestrous non-territorial solitary carnivores which operate a similar social and spatial structure.
